# Aniline – Addendum: Re-evaluation of the BAT value and evaluation of a pregnancy risk group for the BAT value: Assessment Values in Biological Material – Translation of the German version from 2025

**DOI:** 10.34865/bb6253e10_1ad

**Published:** 2025-03-31

**Authors:** Britta Brinkmann, Rüdiger Bartsch, Sandra Michaelsen, Gerlinde Schriever-Schwemmer, Hans Drexler, Andrea Hartwig, MAK Commission

**Affiliations:** 1 Institute of Applied Biosciences. Department of Food Chemistry and Toxicology. Karlsruhe Institute of Technology (KIT) Adenauerring 20a, Building 50.41 76131 Karlsruhe Germany; 2 Friedrich-Alexander-Universität Erlangen-Nürnberg. Institute and Outpatient Clinic of Occupational, Social, and Environmental Medicine Henkestraße 9–11 91054 Erlangen Germany; 3 Permanent Senate Commission for the Investigation of Health Hazards of Chemical Compounds in the Work Area. Deutsche Forschungsgemeinschaft, Kennedyallee 40, 53175 Bonn, Germany. Further information: Permanent Senate Commission for the Investigation of Health Hazards of Chemical Compounds in the Work Area | DFG

**Keywords:** Anilin, Methämoglobin, Biologischer Arbeitsstoff-Toleranzwert, BAT-Wert, Entwicklungstoxizität, Schwangerschaftsgruppe, aniline, methaemoglobin, biological tolerance value, BAT value, developmental toxicity, pregnancy risk group

## Abstract

The German Senate Commission for the Investigation of Health Hazards of Chemical Compounds in the Work Area (MAK Commission) re-evaluated the data for aniline [62-53-3] to verify the biological tolerance value (BAT value) of 500 μg aniline/l urine and to assign the BAT value to a pregnancy risk group, considering all toxicological end points. Relevant studies were identified from a literature search. The critical effect of aniline exposure in humans is considered to be the formation of methaemoglobin. It must be ensured that the methaemoglobin value remains below 5%, even if the BAT value is maintained. In an experimental study, the methaemoglobin content in human blood rose from 0.72% to an average methaemoglobin value of 1.2% (maximum of 2.07% methaemoglobin) during six hours of exposure to 2 ml aniline/m^3^ (corresponding to the maximum concentration at the workplace, MAK value). With linear extrapolation an excretion of 224 μg aniline/l would be expected at the end of an eight-hour exposure period. Consideration of the increased respiratory volume and the critical methaemoglobin increment results in a concentration of 500 μg aniline (after hydrolysis)/l urine. The BAT value has therefore been confirmed. Due to acute toxic effects, the BAT value must be regarded as the maximum value, i. e. it must be ensured that this value is not exceeded. Sampling should take place at the end of exposure or the end of a shift.

Because the no observed adverse effect concentration (NOAEC) for methaemoglobin content in relation to developmental toxicity in humans is not known, a risk for the unborn child cannot be ruled out, even in cases of compliance with the BAT value. The BAT value is therefore assigned to Pregnancy Risk Group B. As an indication of the prerequisite for an assignment to Pregnancy Risk Group C, a concentration of 30 µg aniline/l urine has been calculated from the lowest value of the natural background range of mean methaemoglobin values in (pregnant) women. At this urinary aniline concentration, damage to the embryo or foetus is unlikely.

**Table:** 

**BAT value (2015)**	**500 μg aniline (after hydrolysis)/l urine** BAT value derived as ceiling value because of acute toxic effects
	Sampling time: end of exposure or end of shift
Developmental toxicity (2024)	Group B, prerequisite for Group C: 30 µg aniline (after hydrolysis)/l urine
**BLW (2015)**	**100 μg aniline (released from haemoglobin conjugate)/l blood**
	Sampling time: after exposure for at least 3 months

**MAK value (1983)**	**2 ml/m^3^ ≙** **7.7 mg/m^3^**
Developmental toxicity (2024)	Group B

## Re-evaluation

In 2015, based on data from a study by Käfferlein et al. (2014) a BAT value of 500 μg aniline (after hydrolysis)/l urine as a maximum value due to acute toxic effects was derived for aniline (translated in Bolt et al. 2017). The MAK value of 2 ml/m^3^ (translated in Henschler 1993) was assigned to Pregnancy Risk Group B (available in German Hartwig and MAK Commission 2025). As the BAT value was not derived in correlation to the MAK value, an assignment of the substance to a pregnancy risk group has to be evaluated for the BAT value.

## Toxicokinetics and metabolism

Only human data are presented. Data on toxicokinetics in animals have been extensively described by Bolt et al. 2017; Hartwig 2010; Hartwig and MAK Commission 2019 a).

### Half-life of methaemoglobin

At severe exposures from aromatic amino or nitro compounds with considerably increased methaemoglobin values (intoxications), the substance has a half-life of up to 12 hours (Bolyai et al. 1972; Leng and Bolt 2008). After inhalation exposure (aniline) in rats, the half-life of methaemoglobin was found to be 75 minutes (Hartwig 2010; Kim and Carlson 1986).

A study with 19 individuals exposed over a period of 6 hours via inhalation to 2 ml aniline/m^3^ (Käfferlein et al. 2014), suggests that the half-life of methaemoglobin is around 3 to 4 hours, whereby its initial half-life can be given as about 2 hours. Accumulation over the course of a workweek is not to be expected.

It has been shown that aniline can pass through the placental barrier in pregnant rats. Within an hour of subcutaneous administration (unknown dosage) to the mother rats, more aniline was found in the foetal plasma than in the maternal plasma. At about 1.5 hours, the half-life in blood was the same for both mother and foetus (Henschler 1990; Maickel and Snodgrass 1973).

## Background exposure: aniline

In 856 non-smokers and 145 smokers, for non-smokers a median of 2.99 µg aniline/l urine and a 95^th^ percentile of 14.49 µg aniline/l urine (range < 0.1–384 µg/l) and for smokers a median of 3.28 µg aniline/l urine and a 95^th^ percentile of 13.46 µg aniline/l urine (range 0.19-35.71) were determined (Kütting et al. 2009).

In 81 non-smokers from the general population (48 women, 33 men; age 20-61 years) a median of 1.88 µg aniline/l, a 95^th^ percentile of 10.9 µg aniline/l, and a maximum of 130 µg aniline/l urine were given (Seidel 2005). 

### Pregnant women

In a Brazilian study of 300 pregnant women (244 non-smokers, 56 smokers) on the determination of aromatic amines in urine, the following concentrations of aniline in urine were reported: median: 1.38 µg aniline/l, 25^th^ percentile: 0.89 µg aniline/l, 75^th^ percentile: 2.87 µg aniline/l, minimum: 0.48 µg aniline/l, maximum: 218.84 µg aniline/l urine (Souza et al. 2023).

In the USA, 45 chemicals were measured in the urines of 171 pregnant women between 2008 and 2020. Aniline was detected in every urine sample; the concentrations were as follows: geometric mean: 0.81 µg/l, median: 0.74 µg/l, 25^th^ percentile: 0.52 µg/l, 75^th^ percentile: 1.1 µg/l, minimum: 0.15 µg/l, maximum: 34 µg aniline/l urine (Choi et al. 2022).

## Background exposure: methaemoglobin

Background exposure with methaemoglobin in the non-exposed general population was reported as follows: mean: 0.78%, standard deviation: 0.37%, median: 0.85%, 95^th^ percentile: 1.28% (ACGIH 2006). An upper limit of 1.5% methaemoglobin could be established. The physiological background range for methaemoglobin concentrations is reported on average as < 1%. An increased methaemoglobin concentration of more than 1.5% is recognized as an exposure marker in humans, indicating exposure to methaemoglobin-forming substances (Leng and Bolt 2008).

In a volunteer study, 9 women and 10 men were exposed to 2 ml aniline/m^3^ over a period of 6 hours. The original data on methaemoglobin formation in the 9 female subjects are presented in Table 1 (Käfferlein 2023). At the start of exposure, the mean value for methaemoglobin in the 9 women was determined to be 0.75% (range: 0.53–0.97%). In addition, methaemoglobin concentrations were determined in 8 control subjects over a period of 5 days, whereby the mean value was found to be 0.58 ± 0.15% (range: 0.2–1%) and the 95^th^ percentile was 0.8%. The range of fluctuations was reported as 21% within a day and as 29% within 5 days (Käfferlein et al. 2014). Since the test subjects were only exposed under a breathing volume of 30 l/min on a bicycle ergometer for one sixth of the exposure time, this corresponds to an average breathing volume of 12.5 l/min if a resting breathing volume of 9 l/min is taken as a basis. The data show that, after six-hour inhalation exposure to 2 ml/m^3^, extrapolated to 8 hours and taking increased respiratory volume at the work place into account (21 l/min), a level of 1.5% methaemoglobin would be exceeded in eight of the nine women. After 6 hours of exposure to 2 ml aniline/m^3^ the average urine concentration for all 9 women was determined to be about 150 µg aniline/l (see Table 2).

**Tab. 1:** Methaemoglobin concentrations [%] and increments of 9 women exposed to 2 ml aniline/m^3^ for 6 hours (Käfferlein 2023)

Subject^a)^	Time [h]										MetHb^d)^	MetHb^e)^
	0	2^c)^	4^c)^	6^c)^	7	8	10	12	24	48		
1 slow	0.70	1.20	1.70	2.07	–	1.40	0.97	1.00	0.60	0.83	2.52	**3.76**
2 rapid	0.70	1.05	1.13	1.27	–	0.87	0.70	0.83	0.50	0.90	1.46	**1.97**
3 slow	0.97	0.90	0.95	1.30	1.30	0.77	1.00	1.03	1.10	0.77	1.41	**1.71**
4 slow	0.85	0.90	0.97	1.33	1.00	0.57	0.83	0.87	0.50	0.57	1.49	**1.93**
5 rapid	0.80	0.83	1.00	1.07	0.80	0.67	0.73	0.63	0.53	0.45	1.16	**1.40**
6 slow	0.63	0.80	0.77	0.90	0.70	0.67	0.75	0.60	0.63	0.53	0.99	**1.23**
7 slow	0.93	0.93	1.07	1.30	1.00	1.00	0.87	0.70	0.73	0.67	1.42	**1.75**
8 slow	0.53	0.73	0.87	1.03	0.80	0.83	0.80	0.70	0.67	0.60	1.20	**1.65**
9 slow	0.60	0.80	0.97	1.03	0.77	0.60	0.73	0.70	0.60	0.40	1.18	**1.57**
**Mean**	**0.75**	**0.82**	**1.05**	**1.26**	**0.91**	**0.82**	**0.82**	**0.79**	** 0.65**	** 0.64**	**1.43**	**1.89**
**Mean (increment^b)^)**				**0.51**	**0.16**	**0.07**	**0.07**	**0.04**	**–0.1**	**–0.11**		

^a)^ acetylator status

^b)^ increment: difference of methaemoglobin concentration at timepoint 0 h

^c)^ under exposure to 2 ml aniline/m^3^

^d)^ linear extrapolated to 8 h of exposure of increment 0–6 h

^e)^ increased respiratory volume considered for the increment

MetHb: methaemoglobin

**Tab. 2:** Aniline concentrations in urine and methaemoglobin increments [%] in blood of 9 women exposed to 2 ml aniline/m^3^ for 6 hours (Käfferlein 2023, 2024)

Subject^a)^	Aniline [µg/l urine]	Aniline [µg/l urine]	MetHb increment	Aniline [µg/l urine]	MetHb increment	Aniline [µg/l urine]	MetHb increment
	pre-exposure	after 2 h exposure		after 4 h exposure		after 6 h exposure	
1 slow	6.67	118.35	0.5	256.21	1	343.49	1.37
2 rapid	7.48	53.68	0.35	174.03	0.43	107.33	0.57
3 slow	4.86	53.37	-0.07	129.33	-0.02	163.27	0.33
4 slow	4.50	45.77	0.05	70.05	0.12	79.5	0.48
5 rapid	5.67	57.17	0.03	72.29	0.2	92.31	0.27
6 slow	1.51	51.52	0.17	117.62	0.14	95.82	0.27
7 slow	1.24	50.76	0	101.5	0.14	173.97	0.37
8 slow	7.32	147.5	0.2	183.77	0.34	199.12	0.5
9 slow	5.95	53.24	0.2	110.08	0.37	96.96	0.43
**Mean**	**5.02**	** 70.18**	** 0.16**	**134.99**	** 0.3**	**150.2**	**0.51**

^a)^ acetylator status

h: hours; MetHb: methaemoglobin

In a longitudinal study, the influence of nitrate-containing drinking water on the methaemoglobin concentrations was investigated in up to 357 pregnant women (see Table 3). Depending on the respective week of pregnancy, the mean values lied in a range of 0.39–0.74% (maximum value of 3.6%). The study found a relatively low number of pregnant women (2 to 5) with methaemoglobin concentrations above the physiological upper limit of 2% defined by Wright et al. (1999). Mean methaemoglobin concentrations decrease during pregnancy, even when accounting for certain covariates. The influence of vitamins on low methaemoglobin values has been discussed. It is not described whether there were complications during pregnancy or effects in the newborns (Manassaram et al. 2010).

**Tab. 3:** Summary of methaemoglobin background values

Collective	Methaemoglobin [%] mean ± SD	Range	Fluctuation range 21% (within 1 day)/ 29% (within 5 days)	Reference
9 ♀	0.75	0.53–0.97	0.16/0.22%	Käfferlein et al. 2014
1 ♀	0.27	–	–	
1 ♀	0.03	–	–	
8 control subjects (no more information) (non-smokers, 5 days)	0.58 ± 0.15 0.8 (95^th^ percentile)	0.2–1	0.12/0.17%	
Pregnant women:				Manassaram et al. 2010
357 (initial study)	0.74 ± 0.48	0.1–2.2; n = 2^a)^	0.16/0.22%	
317 (PW 20)	0.67 ± 0.52	0.1–3.6; n = 3^a)^	0.14/0.19%	
316 (PW 28)	0.58 ± 0.46	0.1–2.1; n = 2^a)^	0.12/0.17%	
304 (PW 36)	0.51 ± 0.46	0.1–2.2; n = 2^a)^	0.11/0.15%	
300 (on date of delivery)	0.42 ± 0.47	0.1–2.3; n = 2^a)^	0.09/0.12%	
295 (2–4 weeks post-delivery)	0.39 ± 0.51	0.1–3.0; n = 5^a)^	0.08/0.11%	
10 pregnant ♀ without pregnancy complications	1.3 ± 0.9	0.4–2.8; n = 3^a)^	0.27/0.38%	Tabacova et al. 1997

^a)^ with > 2% methaemoglobin

PW: week of pregnancy; SD: standard deviation

## Polymorphism of methaemoglobin reductase

The reduction of methaemoglobin to haemoglobin is catalysed by the soluble NAD(P)H-dependent cytochrome b5 reductase 3 (gene: *CYB5R3*) in the erythrocyte cytoplasm. As an electron acceptor, cytochrome b5 type A (gene: *CYB5A*), which is membrane-bound to the endoplasmic reticulum, is also necessary for the reaction (Percy and Lappin 2008). In rare congenital methaemoglobinaemia, mutations in the *CYB5R3* gene are present. To date, over 80 distinct pathogenic variants of the *CYB5R3* gene have been reported. Based on the severity of the enzyme defect, two phenotypes for autosomal recessive hereditary methaemoglobinaemia are distinguished. In type I, a missense variant only leads to an instable enzyme in the erythrocytes. Cyanosis arises starting at birth and is associated with mild symptoms such as headache, weakness, and exertional dyspnoea, and is generally well tolerated. Methaemoglobin levels of up to 30% may occur. Most patients do not experience any symptoms. The type I phenotype occurs at a frequency of 1:1000 in certain isolated populations. In type II, a low expression rate or low activity of cytochrome b5 reductase arises in all tissues, which leads to changes in lipid metabolism as well as to neurological impairments. The methaemoglobin level lies in the range of 8–40%. Due to neurological disturbances (opisthotonus, axial hypotension, variable dystonia, choreoathetoid movements), microcephaly, and retarded growth, type II methaemoglobinaemia exhibits a significantly more severe disease progression. These effects begin to develop in the ninth month of life. The mortality rate prior to reaching adulthood is very high. A lack of *CYB5R* has been detected in specific population groups of the USA and Russia. A selection advantage of the Africa-specific polymorphism *CYB5R3-Thr117Ser*, which has been detected in 46% of African Americans, may offer protection against malaria (Iolascon et al. 2021).

## Re-evaluation of the BAT value

The formation of methaemoglobin is recognised as a critical effect of aniline in humans. Based on the reference-value concept, an increase in methaemoglobin concentrations of more than 1.5% is considered an exposure marker in humans and should not be understood as a health-based value. An increase of over 1.5% indicates exposure to methaemoglobin-forming substances. Adverse health effects from methaemoglobin are not to be expected in healthy adults with a methaemoglobin concentration of up to 5% (Leng and Bolt 2008).

The experimental study (9 women and 10 men) by Käfferlein et al. (2014) shows that after six-hour exposure to 2 ml aniline/m^3^ (via inhalation; dermal exposure was largely ruled out), the methaemoglobin content in blood of 0.72% rose to a mean methaemoglobin value of 1.2%. A value of 2.07% was measured as the highest individual methaemoglobin value. The maximum value of the aniline-induced methaemoglobin increment was found to be 1.35% (2.07% − 0.72% = 1.35%). No plateau was reached, such that a linear extrapolation to eight hours was conducted. The increment of the maximum value was thereby 1.8% (1.35% × 8/6) after eight hours. Considering increased respiratory minute volume in the workplace (10 m^3^) yields a methaemoglobin increment of 3% (21/12.5 l/min = 1.68; 1.8% × 1.68 = 3%). It is important to ensure compliance with the BAT value of less than 5% methaemoglobin, even in individual cases. Under experimental conditions of aniline exposure at the level of the MAK value, the determined 8h increment is lower by a factor of 1.33 (4%:3%) when compared to a maximum allowable methaemoglobin increment of 4%, ascertained under consideration of the background exposure of < 1% methaemoglobin. After six-hour exposure, the mean value for aniline excretion was found to be 168 µg/l; at the end of an eight-hour exposure period, an excretion of 224 µg/l would therefore be expected. Taking increased respiratory volumes into account (21/12.5 l/min = 1.68; 224 µg/l × 1.68) yields a concentration of 376 µg aniline/l urine. Assuming a factor of 1.33 yields an expected excretion of 500.5 µg aniline/l urine. This value, determined under consideration of increased respiratory volume, 

**confirms the BAT value of 500 µg aniline (after hydrolysis)/l urine.**

The BAT value refers to a methaemoglobin value of 5% and should be regarded as a maximum value, which means that it must be ensured that this value is not exceeded. Sampling should take place at the end of exposure or the end of a shift.

## Developmental toxicity 

### Humans

#### Methaemoglobin formation as the most sensitive effect

Methaemoglobin values were determined in 61 pregnant women, independent of the age of the mother, gestational age, or smoker status. In the 10 women with normal pregnancies, methaemoglobin values lied between 0.4 and 2.8%. In total, 64% of the methaemoglobin values exceeded the physiological upper limit of 2% indicated by the authors. Pregnancy complications such as anaemia (< 10 g haemoglobin/dl), premature birth, sepsis, proteinuria, high blood pressure, and preeclampsia were observed in cases of increased methaemoglobin values up to 11.2%. These effects were attributed to cellular damage, increased lipid peroxidation, and a decrease in maternal antioxidative reserves. The authors explain that the increased methaemoglobin values indicate high exposure to nitrite and nitrate as well as other methaemoglobin-forming substances (Tabacova et al. 1997). 

In another study from this working group, methaemoglobin concentrations and antioxidative capacity were determined in maternal and umbilical-cord blood from 51 mother-child pairs on the date of the child’s birth. The study was conducted due to high concentrations of NO_2_ in the ambient air (mean value of 23.1 µg/m^3^, peaks up to 238.5 µg/m^3^) as well as high nitrate concentrations in drinking water and vegetables. An evaluation was performed according to birth weight (normal and low (< 2500 g)), birth before week 37 of pregnancy, and “foetal distress”. Smokers comprised 37% of the sample, and 8 mothers experienced anaemia during pregnancy. Pregnancy progressed without complications and with normal birth weights for 36 mothers, 6 mothers experienced a premature birth, birth weight was low for 7 newborns despite a normal duration of pregnancy, one newborn was breech and another newborn was delivered by Caesarean section. In maternal blood, 55% of methaemoglobin values exceeded 2% and, in umbilical-cord blood, this value exceeded 2.8%. Methaemoglobin values in maternal and umbilical-cord blood were higher for those with abnormal birth outcomes. The mean values for maternal methaemoglobin concentrations were about twice as high for mothers with premature births and “foetal distress” compared to mothers who delivered normally. Mean methaemoglobin values in umbilical-cord blood were four times as high for premature births compared to normal births, whereby a maximum value of 34% was observed. The mean methaemoglobin value in newborns with low birth weights was about 1.5 times higher than that of newborns with normal birth weights, and the highest measured value was 16%. Smoking during pregnancy was not associated with increased methaemoglobin values in either maternal or umbilical-cord blood, although the mean birth weight for newborns from smokers was less than that of non-smokers (3046 g compared to 3197 g). Moreover, smokers had a higher rate of premature births (12% or 6%). Statistically significant decreases in antioxidative capacity were observed in cases of increased methaemoglobin concentrations in maternal and newborn blood (Tabacova et al. 1998). 

In an environmental study by Mohorovic (2003), the correlation between ambient SO_2_ concentrations and methaemoglobin values was investigated in 260 pregnant women. The range of methaemoglobin concentrations can be interpreted from a figure as 1.2–2 g/l (Mohorovic 2003). Since no detailed results for methaemoglobin values were reported, this study was not considered.

### Differences in sensitivity between humans, rats, and foetuses/newborns

Foetal haemoglobin is more easily oxidised than that of adults. The activity of methaemoglobin reductase, which is responsible for the regeneration of functional haem from methaemoglobin, shows considerable differences between species and is age-dependent (Klimmek et al. 1988; Power et al. 2007; Rockwood et al. 2003). In the erythrocytes of rats and mice, respectively, the activity of the NADH-dependent reductase is about 5 and 10 times higher than that of humans (EU 2004; Hartwig and MAK Commission 2019 b; Smith 1986). In an investigation of various species with both adult animals and newborns as well as humans (3 adults, 2 newborns), methaemoglobin-reductase activity was about twice as high in adult humans compared to that of newborns. In rats, (3 adults, 16 newborns) and mice (4 adults, 8 newborns), activity was considerably higher in newborns (Lo and Agar 1986). Due to their lower levels of methaemoglobin-reductase activity, human newborns are therefore much more sensitive to methaemoglobin-forming substances than human adults (Power et al. 2007).

Methaemoglobin formation, degeneration, and erythrocyte toxicity combine into a complex, multistep process, whereby the availability of antioxidative substances plays an important role (Pauluhn 2004). In human erythrocytes, the activity of glutathione reductase is about 5.5 times as high and that of the catalase is about 2.5 times as high as compared to rats (Godin and Garnett 1992). 

## Evaluation of a pregnancy risk group for the BAT value

There are no investigations on the effects experienced by newborns exposed to a methaemoglobin level of up to 5% in utero. In pregnant women, methaemoglobin levels of up to 2% are considered physiological. Foetal haemoglobin is more easily oxidised to methaemoglobin than that of adults. Moreover, methaemoglobin-reductase activity is about twice as high in adults compared to newborns. Due to both effects, newborns react much more sensitively to methaemoglobin-forming substances than adults. The activity of methaemoglobin reductase is about 5 to 10 times as high as in the erythrocytes of rats and mice than in those of humans. Human erythrocytes therefore react more sensitively to methaemoglobin-forming substances, since they reduce methaemoglobin much more slowly. For this reason, reproduction toxicity should therefore not be evaluated from animal experiments.

The individual maximum value measured by Käfferlein et al. (2014) was 2.07% methaemoglobin after six-hour inhalation exposure to 2 ml aniline/m^3^. As previously described, the methaemoglobin increment from aniline would be 3% at an increased respiratory minute volume of 21 l. The authors note that the respiratory minute volumes of pregnant women increase by about 20–50% after the end of the first trimester, whereby it does not, however, exceed the presumed respiratory minute volume of 21 l during physical labour.

Since the no observed adverse effect concentration (NOAEC) for methaemoglobin concentrations with respect to developmental toxic effects in humans is not known, and because a foetus reacts much more sensitively than an adult, risk to an unborn child cannot be ruled out, even in cases of compliance with the BAT value of 500 µg aniline/l urine; for this reason, 

**the BAT value of aniline is assigned to Pregnancy Risk Group B.**

 In support of this decision, the reader is referred to the assignment of dichloromethane and carbon monoxide to Pregnancy Risk Group B based on a CO‑Hb value of 5%.

### Prerequisite for assignment to Pregnancy Risk Group C

**Fig. 1:**
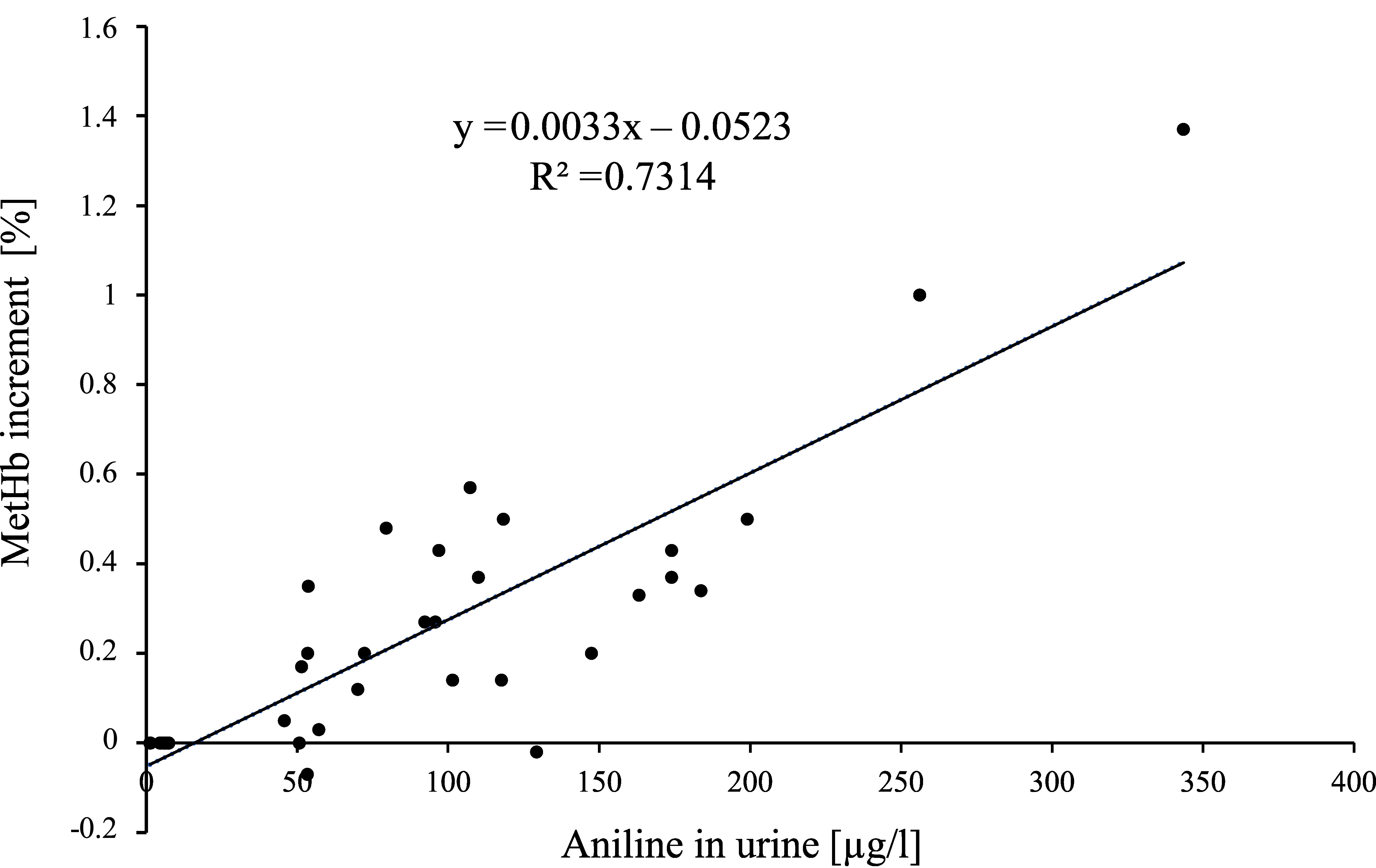
Regression equation determined from the individual values of the aniline concentration in the urine and the methaemoglobin increments of the 9 women after 2-, 4- and 6-hour exposure to 2 ml aniline/m^3^

**Fig. 2:**
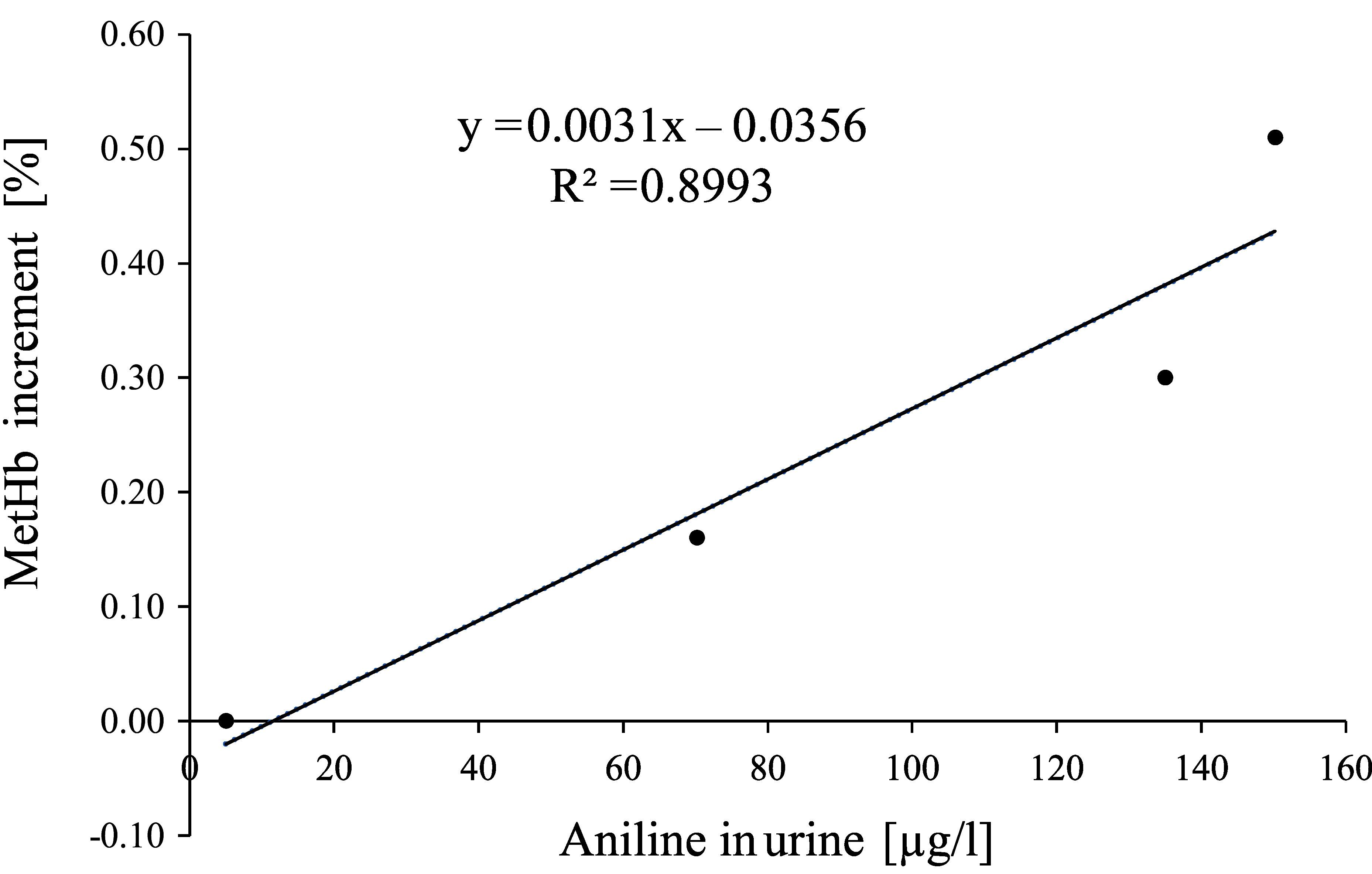
Regression equation determined from the mean values of the aniline concentration in the urine and the methaemoglobin increments of the 9 women after 2-, 4- and 6-hour exposure to 2 ml aniline/m^3^

The mean physiological background values for the percentage of methaemoglobin is below 1% for women, in a range of 0.03 to 0.97%. For pregnant women, mean background values of 0.39 to 1.3% methaemoglobin (range of 0.1–3.6%) have been reported (see Table 3). The analysis of a methaemoglobin value on a percentage basis (MetHb%) in pregnant women shows a rather large range of fluctuations. The range of fluctuations includes, however, measurement inaccuracy, such that the actual range of fluctuations for methaemoglobin values is probably lower. In the study by Käfferlein et al. (2014), fluctuation ranges of 21% within one day and 29% within five days are reported. These fluctuation ranges correspond to increments of a minimum of 0.08 and a maximum of 0.38% methaemoglobin over one or five days, respectively, of the mean methaemoglobin values of (pregnant) women (see Table 3). If a urinary aniline concentration causes a methaemoglobin increment within the natural fluctuation range of methaemoglobin levels, no adverse effects on the unborn child are to be expected. To determine these aniline concentrations, regression analysis is used which is ascertained from the mean and/or individual values for urinary aniline concentrations as well as the methaemoglobin increments of 9 women after 2-, 4-, and 6-hour exposure (see Figure 1 and 2).

Methaemoglobin increments of 0.08 and 0.38% would correspond to aniline concentrations of 37.29 and 134.06 µg/l urine (calculated from the regression equation based on the mean values: y = 0.0031x − 0.0356 (r^2^ = 0.8993)) or 40.1 and 131 µg/l urine (calculated from the regression equation based on the individual values: y = 0.0033x – 0.0523 (r^2^ = 0.7314)). Since foetuses are much more sensitive to methaemoglobin-forming substances than adults, the lowest value of the methaemoglobin increment is used for the derivation of a concentration as a prerequisite for Pregnancy Risk Group C.

As a **prerequisite for assignment to Pregnancy Risk Group C**, a concentration of

**30 µg aniline/l urine**

is established. Developmental toxicity is unlikely at this concentration.
